# Necrotic Cells from Head and Neck Carcinomas Release Biomolecules That Are Activating Toll-like Receptor 3

**DOI:** 10.3390/ijms242015269

**Published:** 2023-10-17

**Authors:** Tea Vasiljevic, Marko Tarle, Koraljka Hat, Ivica Luksic, Martina Mikulandra, Pierre Busson, Tanja Matijevic Glavan

**Affiliations:** 1 Laboratory for Personalized Medicine, Division of Molecular Medicine, Rudjer Boskovic Institute, Bijenicka 54, 10000 Zagreb, Croatia; 2 Department of Maxillofacial Surgery, Dubrava University Hospital, School of Medicine, University of Zagreb, Gojko Šušak Avenue 6, 10000 Zagreb, Croatia; tarlemarko1@gmail.com (M.T.);; 3 School of Dental Medicine, University of Zagreb, Gunduliceva 5, 10000 Zagreb, Croatia; 4 Division of Oncology and Radiotherapy, University Hospital for Tumors, Sestre Milosrdnice University Hospital Center, Vinogradska Cesta 29, 10000 Zagreb, Croatia; 5 CNRS-UMR 9018-METSY, Gustave Roussy Institute, Université Paris-Saclay, 39 rue Camille Desmoulins, 94805 Villejuif CEDEX, France

**Keywords:** toll-like receptor 3, endogenous ligands, head and neck cancer, exosomes, HEKBlue cells

## Abstract

Tumor necrosis is a recurrent characteristic of head and neck squamous cell carcinomas (HNSCCs). There is a need for more investigations on the influence of biomolecules released by these necrotic foci in the HNSCC tumor microenvironment. It is suspected that a fraction of the biomolecules released by necrotic cells are damage-associated molecular patterns (DAMPs), which are known to be natural endogenous ligands of Toll-like receptors (TLRs), including, among others, proteins and nucleic acids. However, there has been no direct demonstration that biomolecules released by HNSCC necrotic cells can activate TLRs. Our aim was to investigate whether some of these molecules could behave as agonists of the TLR3, either in vitro or in vivo. We chose a functional approach based on reporter cell exhibiting artificial TLR3 expression and downstream release of secreted alkaline phosphatase. The production of biomolecules activating TLR3 was first investigated in vitro using three HNSCC cell lines subjected to various pronecrotic stimuli (external irradiation, serum starvation, hypoxia and oxidative stress). TLR3 agonists were also investigated in necrotic tumor fluids from five oral cancer patients and three mouse tumor grafts. The release of biomolecules activating TLR3 was demonstrated for all three HNSCC cell lines. External irradiation was the most consistently efficient stimulus, and corresponding TLR3 agonists were conveyed in extracellular vesicles. TLR3-stimulating activity was detected in the fluids from all five patients and three mouse tumor grafts. In most cases, this activity was greatly reduced by RNAse pretreatment or TLR3 blocking antibodies. Our data indicate that TLR3 agonists are consistently present in necrotic fluids from HNSCC cells and mainly made of dsRNA fragments. These endogenous agonists may induce TLR3, which might lead to a protumorigenic effect. Regarding methodological aspects, our study demonstrates that direct investigations—including functional testing—can be performed on necrotic fluids from patient tumors.

## 1. Introduction

Head and neck squamous cell carcinomas (HNSCCs) account for one-sixth of all human malignancies worldwide. They represent a major public health problem. Alcohol and tobacco abuse are among the main etiological factors [1]. However, a fraction of HNSCCs is related to human papillomavirus infections, especially among oropharyngeal and tonsil carcinomas [2]. Anoxia and necrosis are more common and severe in HNSCCs than in many other categories of human malignancies [3,4].

Cell necrosis is defined as an unprogrammed form of cell death that occurs in response to overwhelming chemical or physical distress. The occurrence of necrotic cells is one of the most consistent characteristics of all human solid malignancies [5]. To a large extent, it is linked to the quantitative and qualitative mismatch between tumor angiogenesis and the proliferation of malignant cells. Insufficient vascularization results in acute shortages of oxygen and nutrients, inducing the formation of tumor necrotic foci of various sizes. Necrotic cells undergo cell lysis and release various types of biomolecules, either in native or altered configurations. A number of these diffusible necrotic biomolecules induce changes in the phenotype and behavior of distinct living cells with the status of target cells (for a review, see [6]). Many of these changes result from the binding of necrotic biomolecules to Toll-like receptors (TLRs) expressed by the target cells.

Initially described as actors associated with embryonic development in invertebrates, TLRs were later recognized as important players in the innate immune response in mammals. They can be found in immune cells, most often in dendritic cells and macrophages [7]. Nowadays, they are known to be expressed in a wide range of tissues and to play a role in the homeostasis of these tissues [8]. They are also often expressed by malignant cells in various types of tumors [9]. TLRs belong to a larger protein family called pattern recognition receptors (PRRs). Each of them recognizes a specific category of molecular motifs often shared by biomolecules derived from several groups of pathogens. Those motifs belong to bacteria, viruses, parasites or fungi and are called pathogen-associated molecular patterns (PAMPs). Besides PAMPs, which are externally derived molecules, TLRs also recognize damage-associated molecular patterns (DAMPs) that have an endogenous origin, for example, tissue damage and/or cell death. Both PAMPs and DAMPs can directly activate TLR signaling pathways [10,11]. Stimulation of TLR receptors by their ligands activates different signaling pathways and transcription factors: Nuclear factor kappa-light-chain-enhancer of activated B cells (NF-κB), Interferon regulatory factor 3 (IRF3), Interferon regulatory factor 7 (IRF7), Activator protein 1 (AP-1) and cAMP-response element binding protein (CREB) [12]. Today, 10 TLRs have been recognized in humans: TLR1, 2, 4, 5, 6, 10 and 11 reside, to a large extent, on the plasma membrane, whereas TLR3, 7, 8 and 9 are mostly found in endosomes and the endoplasmic reticulum [7]. Among DAMPs activating TLRs, we can mention heat shock proteins (HSPs) and High mobility group box 1 (HMGB1) for TLR2; HSPs, fibrinogen, heparan sulfate, fibronectin, hyaluronic acid and HMGB1 for TLR4; self ssRNA for TLR7; and self-DNA for TLR9 [13].

Like other TLRs, TLR3 can be activated by PAMPs and DAMPs. Pieces of double-strand RNA (dsRNA) of minimal size (40–50 bp) are the most well-known ligands of TLR3. Under physiological conditions, these dsRNAs are rare inside the cells and circulate through intracellular routes distinct from those of TLR3. In contrast, they are abundant and can intersect the routes of TLR3 during viral infections. TLR3 can also be activated by synthetic analogs of dsRNAs such as Polyinosinic:polycytidylic acid (poly(I:C)) or Polyadenylic–polyuridylic acid (poly(A:U)). Kariko et al. and Cavassani et al. previously reported indirect evidence that TLR3 ligands can originate from necrotic cells in vivo [14,15].

In this context, the first aim of our study was to explore whether in vitro exposition of HNSCC cells to various types of stress conditions could induce the release of TLR3-activating DAMPs. The next aim was to determine whether these TLR3-related DAMPs were contained in exosomes. Finally, we wanted to provide evidence that the same type of DAMPs was present in necrotic fluids obtained from mouse tumor models and oral cancer clinical specimens.

## 2. Results

### 2.1. Under In Vitro Stress Conditions, HNSCC Cells Release Endogenous Ligands That Activate TLR3 Reporter Cells

**Figure 1:**
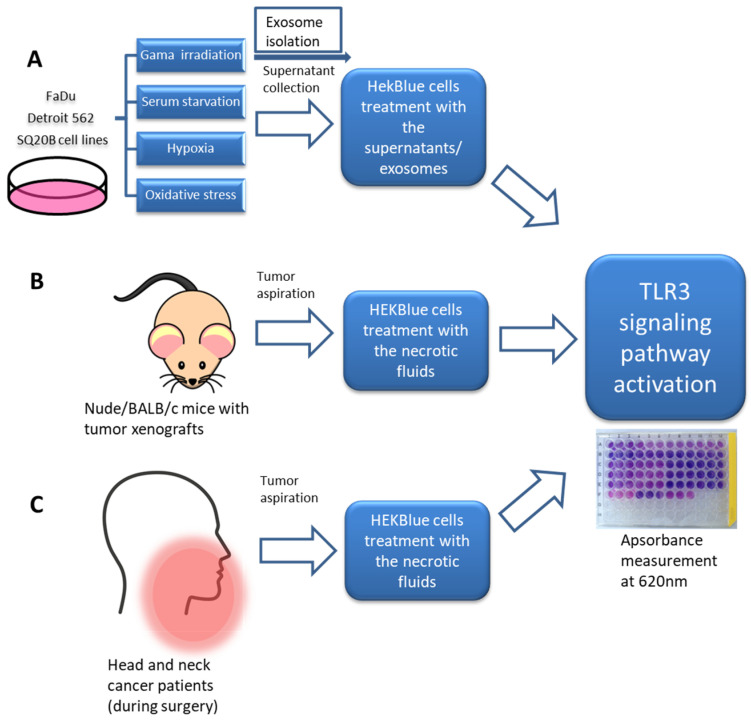
Flow chart showing the experimental design of the study. Conditioned media were collected from cells stressed with irradiation, serum starvation, hypoxia and oxidative stress. In addition, extracellular vesicles were isolated from conditioned media of irradiated cells. Both crude conditioned media and isolated extracellular vesicles were used to stimulate HEKBlue cells in order to measure the activation of the TLR3 signaling pathway (**A**). Additionally, necrotic fluids were collected from tumor grafts carried by nude or Balb/C mice (**B**) and from tumor tissues of patients bearing head and neck carcinomas (**C**). These fluids were also tested on HEKBlue cells in order to measure the activation of the TLR3 signaling pathway.

To determine whether endogenous ligands released from head and neck carcinoma cells exposed to different stressors can activate TLR3, we used the HEKBlue-TLR3 reporter cell line (Invivogen). It was produced by stable co-transfection of the human *TLR3* gene into HEK293 cells, along with an inducible *SEAP* (secreted embryonic alkaline phosphatase) reporter gene. The *SEAP* gene was placed under the control of the IFN-β minimal promoter fused to five NF-κB- and AP-1-binding sites. Stimulation with artificial or natural TLR3 ligands activates NF-κB and AP-1 signaling pathways, which upregulate the production of SEAP. Conditioned culture media were collected from three HNSCC cell lines (Detroit 562, FaDu and SQ20B) following challenges by irradiation, oxidative stress (H_2_O_2_), serum deprivation or hypoxia. The capacity of these conditioned media to activate TLR3 was tested using HEKBlue-TLR3 cells as targets. The enzymatic activity of SEAP released by target HEKBlue-TLR3 cells was used as an index of TLR3 activation. Because NF-κB and AP-1 pathways can be activated in target cells through receptors distinct from TLR3, HEKBlue-null cells were used as negative controls. To further clarify our study design and results, a flow chart of this study is presented in Figure 1.

**Figure 2:**
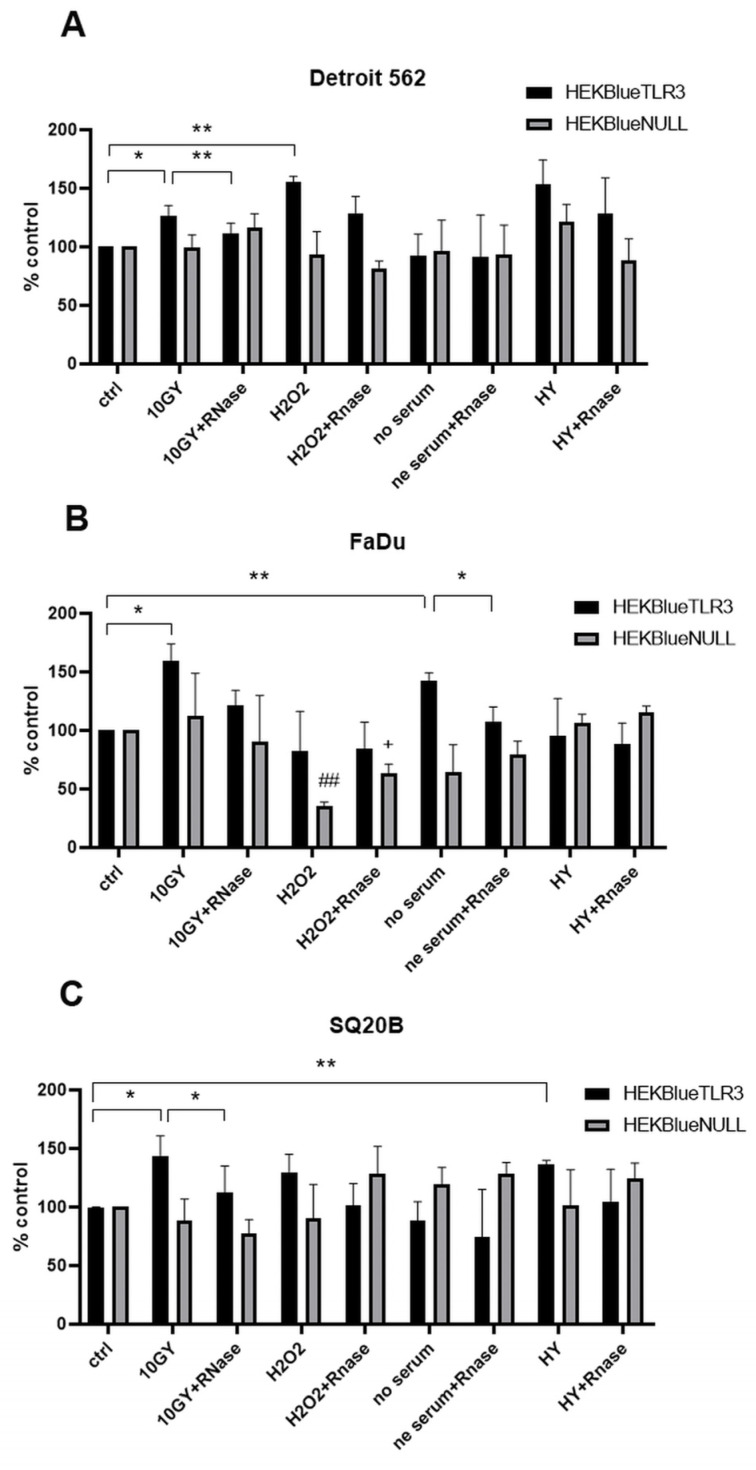
TLR3 activation in HEKBlue-TLR3 and HEKBlue-null reporter cells after treatment with supernatants from Detroit 562 (**A**), FaDu (**B**) and SQ20B cells (**C**) stressed with irradiation, H_2_O_2_, serum deprivation and hypoxia. Ctrl: cells were treated only with fresh culture medium; HY: hypoxia treatment. * *p* < 0.05, ** *p* < 0.01, ## *p* < 0.01 (HEKBlue-TLR3 in relation to HEKBlue-null control), + *p* < 0.05. (HEKBlue-null in relation to HEKBlue-null control). Statistical significance was determined by *t*-test.

As shown in Figure 2, for each cell line, SEAP production was induced in the presence of conditioned medium from unchallenged cells, suggesting a constitutive release of TLR3 ligands by these cells. However, for each cell line, there was an increase in SEAP production by target cells for at least one type of challenge imposed on HNSCC cells: irradiation for all three cell lines, oxidative stress and hypoxia for Detroit 562, serum deprivation for FaDu and hypoxia for SQ20B. No substantial increases in SEAP production were observed when HEKBlue-null cells were exposed to the same supernatants, favoring an increase in the release of endogenous TLR3 ligands by malignant HNSCC cells under various types of stressful conditions, especially external irradiation. For Detroit 562 and SQ20B but not FaDu, a significant decrease in SEAP production was observed when the conditioned media from irradiated cells were treated with RNAse before application on target cells. This strengthens the idea that irradiated HNSCC cells release endogenous TLR3 ligands and suggests that these ligands are—at least in part—made of extracellular RNA fragments. The same reduction in the induction of SEAP production was recorded for conditioned medium from FaDu subjected to serum deprivation (Figure 2).

### 2.2. Endogenous Ligands That Activate TLR3 Reporter Cells Are Contained in Extracellular Vesicles

**Figure 3:**
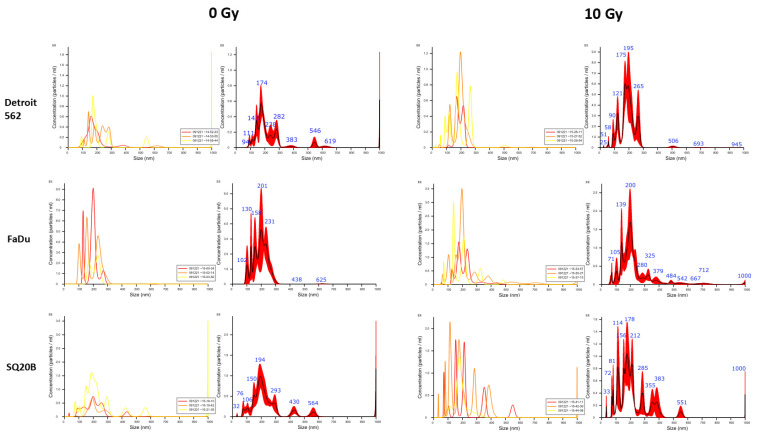
Exosome characterization. Nanoparticle tracking analysis (NTA) of exosomes derived from Detroit 562, FaDu and SQ20B cell supernatants from control non-irradiated cells and cells after irradiation with 10 Gy. The left side shows three runs, with Finite track length adjustment (FTLA) concentration indicated by yellow, orange and red lines, and the graph on the right shows the averaged FTLA concentration for each sample.

**Figure 4:**
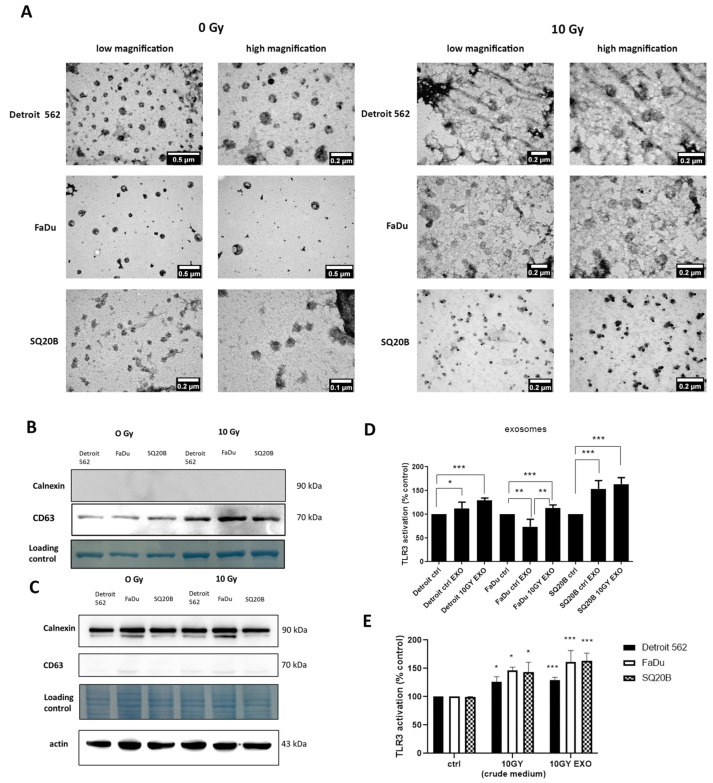
Exosome characterization and the treatment of HEKBlue-TLR3 reporter cells with exosomes. Transmission electron microscopy (TEM) images of exosomes derived from Detroit 562, FaDu and SQ20B cell supernatants from control non-irradiated cells and cells after irradiation with 10 Gy (**A**). Western blot of proteins isolated from exosomes (**B**) and cell lysate. The loading control is the membrane stained with amido black (**C**). TLR3 activation after the treatment of HEKBlue-TLR3 cells with exosomes (800 ng) isolated from control non-irradiated cells and cells after irradiation with 10 Gy (**D**). Comparison between TLR3 activation with crude medium and exosomes isolated after irradiation (**E**). * *p* < 0.05, ** *p* < 0.01, *** *p* < 0.001.

To determine whether endogenous ligands that activate TLR3 reporter cells are contained in extracellular vesicles (EVs), head and neck carcinoma cells were subjected to irradiation, and their extracellular vesicles (mostly exosomes) were isolated from their conditioned media after 48 h of incubation. Irradiation was chosen for this investigation for two reasons: (1) it was the only stimulus inducing an excess of TLR3 activation in all three cell lines and (2) we recently reported that poly(I:C) and cisplatin stimulation leads to radiosensitization of Detroit 562 cells [16]. Exosome size was first checked by Nanoparticle tracking analysis (NTA) (Figure 3). The size of exosomes was 150–200 nm for control and irradiated Detroit 562 cells, 150–300 nm for FaDu and 50–200 nm for SQ20B. Some larger particles—400 to 800 nm in diameter—were observed outside these ranges, especially in SQ20B-conditioned medium, but they were probably exosomes aggregates. Exosome size and morphology was next evaluated by Transmission electron microscopy (TEM) (Figure 4A). Exosome material derived from all three cell types, either control or irradiated, shared similar morphological characteristics: a regular circular shape and a size distribution ranging from 50 to 220 nm, consistent with most literature data. However, there were substantial size variations depending on the cell line, with a range of 80–130 nm for Detroit 562, 160–220 nm for FaDu and only 50–70 nm for SQ20B. To assess the quality of exosome enrichment, we compared protein extracts from whole cells and exosome preparations using Western blots, investigating the amounts of two proteins: one known to be abundant in exosomes (tetraspanin CD63) and the other, calnexin, which is an endoplasmic reticulum marker virtually absent from the exosomes. As expected and as shown in Figure 4B,C, CD63 was detected in exosome preparations but not in whole-cell extracts, whereas the result was the opposite for calnexin.

Using these extracellular vesicles preparations, reporter cells were exposed either to crude-conditioned media or exosomes that were isolated from both unchallenged and irradiated head and neck carcinoma cells. For Detroit 562 and SQ20B cells, using material from unchallenged cells, exposure to exosomes was more efficient than exposure to crude media in the induction of SEAP production, whereas the opposite was observed for FaDu cells. However, when using material from irradiated cells, much greater SEAP production was observed using exosomes in comparison with both crude-conditioned medium and exosomes from unchallenged cells. This result was statistically significant for all three cell lines. Greater responses were recorded for the SQ20B cell line (Figure 4D,E).

### 2.3. Endogenous Ligands Contained in Necrotic Material from Fresh Oral Squamous Cell Carcinoma Activate TLR3 Reporter Cells

**Figure 5:**
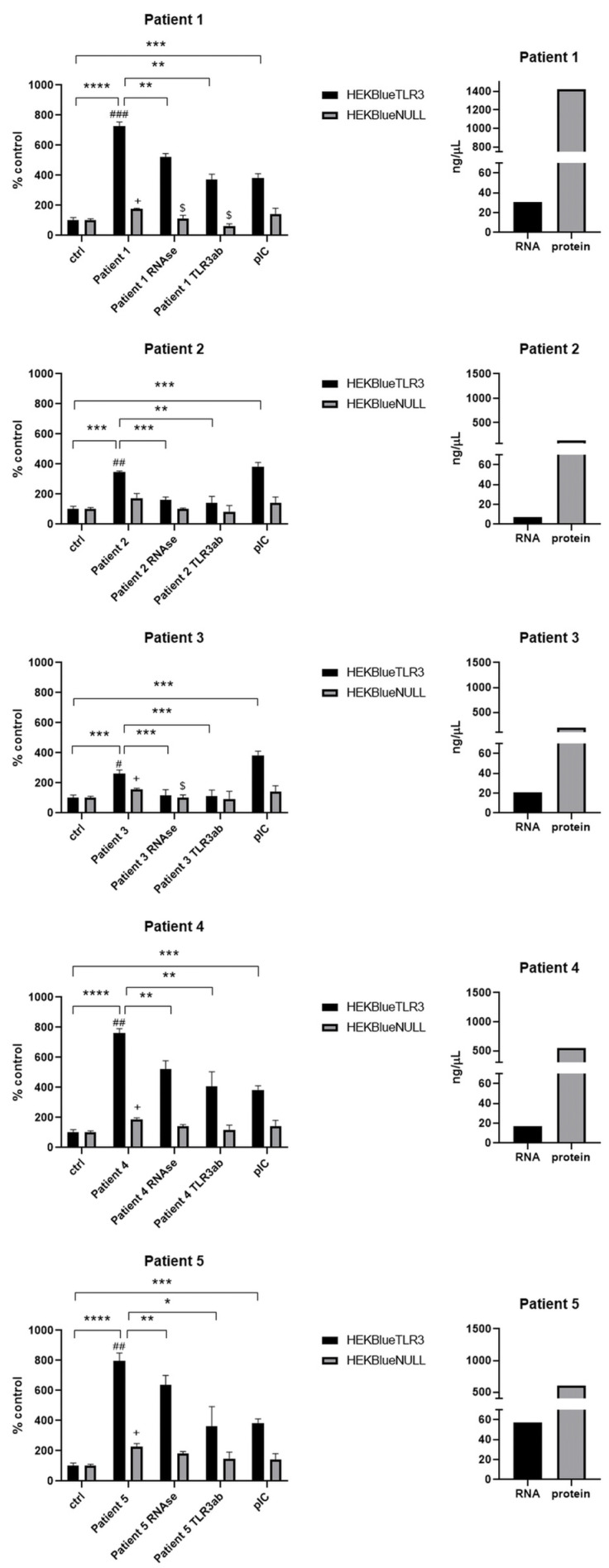
TLR3 activation after the treatment of HEKBlue-TLR3 and HEKBlue-null cells with necrotic fluids derived from oral squamous cell carcinoma patients: patient 1, patient 2, patient 3, patient 4 and patient 5. Ctrl—cells were treated only with fresh culture medium; RNAse—sample pretreated with RNAse; TLR3ab—sample treated with monoclonal TLR3 antibody; pIC—cells treated with 100 ng/mL poly(I:C). The amount of RNA and protein in each sample is also presented. * *p* < 0.05, ** *p* < 0.01, *** *p* < 0.001, **** *p* < 0.001, # *p* < 0.05, ## *p* < 0.01, ### *p* < 0.001 (HEKBlue-TLR3 in relation to HEKBlue-null control). + *p* < 0.05 (HEKBlue-null patient in relation to HEKBlue-null control), $ *p* < 0.05 (HEKBlue-null treatment in relation to HEKBlue-null patient).

Inappropriate angiogenesis, hypoxia and cell necrosis are nearly constant features of solid malignancies in the context of patient tumors, as well as experimental tumors from animal models. Like other investigators in the past, we empirically observed that necrotic material behaves differently from live cells when fragments of clinical tumor specimens or experimental tumors are minced in ordinary culture medium immediately or shortly after ex vivo collection. Cells from necrotic foci lose their cohesion and form a suspension of isolated cells or small aggregates much more easily than live cells. Simultaneously, necrotic fluid resulting from earlier or concomitant cell lysis is released in the medium. No enzymatic treatment is necessary to release this necrotic material, which is generally considered more of an undesirable contaminant. In contrast, for us, rapid tumor mincing was a simple way to prepare fluid samples highly enriched in necrotic material. Such samples were obtained from surgical pieces of oral squamous cell carcinoma for five patients. The amounts of RNA and proteins contained in these samples are also shown in Figure 5. Patients’ clinicopathological data and histopathological features are shown in Table 1. It is important to emphasize that tumor mincing is used not to induce necrosis but to separate the pre-existing necrotic material from the healthy cells.

As shown in Figure 5, all of them induced activation of HEKBlue-TLR3 cells in a statistically significant manner. A certain increase in SEAP production was observed when HEKBlue-null cells were exposed to the same fluids, but it was relatively limited (in the range of 60 to 180% and not exceeding 200%). In contrast, TLR3 activation was at a very high level in comparison with control fluids when the HEKBlue-TLR3 cells were the targets: 728%, 763% and 796% for patients 1, 4 and 5, respectively. In addition, in each case, previous treatments with RNAse or the addition of a TLR3-blocking antibody resulted in a substantial decrease in the activation of HEKBlue-TLR3 cells. All these observations were favor a specific role of the TLR3 pathway.

### 2.4. Endogenous Ligands Contained in Necrotic Material Derived from Experimental Mouse Tumors Activate TLR3 Reporter Cells

**Figure 6:**
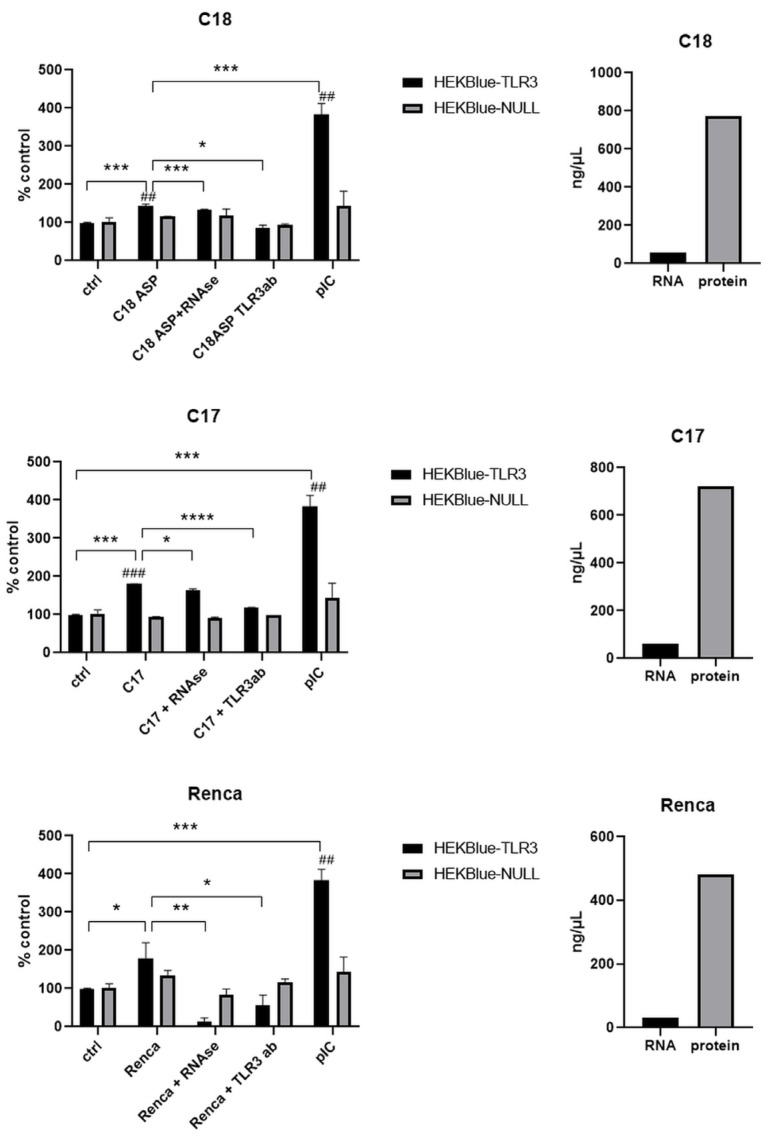
TLR3 activation after the treatment of HEKBlue-TLR3 and HEKBlue-null cells with necrotic fluids derived from different mice models: C18, C17 and Renca. Ctrl—cells treated only with medium; RNAse—sample pretreated with RNAse; TLR3ab—sample treated with monoclonal TLR3 antibody; pIC—cells treated with 100 ng/mL poly(I:C). The amount of RNA and protein in each sample is also presented. * *p* < 0.05, ** *p* < 0.01, *** *p* < 0.001, **** *p* < 0.001, ## *p* < 0.01, ### *p* < 0.001 (HEKBlue-TLR3 in relation to HEKBlue-null control).

Samples of necrotic tumor material were also prepared from mouse tumors either from nude mice xenografts (C17 and C18) or from a syngeneic graft on immunocompetent mice (Renca cells on C57BL/6). Renca cells were used because we were willing to perform investigations on necrotic fluids not only from human tumors xenografted on nude mice but also from murine syngeneic tumor models. However, for technical reasons, it was not possible to use a syngeneic model of HNSCC. Therefore, we resorted to the Renca model, which is available in many laboratories and research institutions. The amounts of RNA and proteins in these samples are also presented in Figure 6.

All these samples induced the activation of HEKBlue TLR3 cells in a statistically significant manner when compared to the crude culture media used as controls. C17 and Renca samples achieved the highest levels of HEKBlue-TLR3 activation (180% and 179%, respectively). C18 activation was 143%. For the C17 and C18 samples, the induction of SEAP production was very low when the HEKBlue-null cells were used as target cells (with a statistically significant difference). In addition, HEKBlue-TLR3 activation was substantially reduced when these fluids were pretreated with RNAse or when they were combined with a TLR3-blocking antibody. For the Renca samples, there was a mild increase in SEAP when HEKBlue-null cells were used as targets. However, there was a dramatic reduction in SEAP production from HEKBlue-TLR3 under RNAse pretreatment or in combination with the TLR3-blocking antibody, confirming a specific contribution of TLR3 in the SEAP response.

## 3. Discussion

It has been reported in previous publications that diffusible biomolecules released from necrotic cells can activate TLR3 in neighboring live cells [14,15]. However, these observations were obtained using experimental systems with limited relevance to cancer physiopathology (mechanical necrosis of HEK293 cells subjected to several freeze–thaw cycles and mouse cecal ligation and puncture, respectively). To the best of our knowledge, we are the first to provide a direct demonstration that necrotic biomolecules released by HNSCC cells can behave as agonists of TLR3 expressed by target living cells. In our experimental setting, in vitro, the release of necrotic biomolecules by malignant HNSCC cells was stimulated by stressing conditions mimicking those imposed on malignant cells undergoing pathological growth in situ (shortage of oxygen or growth factors and oxidative stress) or subjected to treatment procedures (external irradiation). External irradiation was the stimulus that was found to result in the most consistent production of TLR3 agonists, as demonstrated for Detroit 562, FaDu and SQ20B cell lines. We also showed that at least a fraction of the biomolecules released by necrotic HNSCC cells and behaving like TLR3 agonists can be conveyed by extracellular vesicles (EVs). This is consistent with an increasing number of publications suggesting that DAMPs can be found in EVs [17,18,19,20,21,22]. We demonstrated the association of TLR3 agonists with EVs by using external irradiation as a pronecrotic stimulus. In future studies, it will be interesting to investigate whether the same observation can be made using other necrotic stimuli, like hypoxia or oxidative stress. Finally, we showed that biomolecules triggering TLR3 activation are present in necrotic fluids derived from mouse tumor grafts and fresh tumor biopsies collected from patients. Regarding mouse tumor grafts, two were human PDX (patient-derived xenografts) from nasopharyngeal carcinomas (C17 and C18), while the last one, Renca, was not related to HNSCC but is a syngeneic murine tumor of renal origin propagated on immunocompetent mice (C57BL/6). As for C17 and C18, the necrotic biomolecules from Renca were effective on TLR3 reporter cells. Regardless of the category of necrotic biofluids, the evidence of TLR3 involvement was based on the comparison of the reporter response obtained with HEK-TLR3 and HEK-null cells. In addition, for several biofluids from patient or mouse tumor grafts, there was a substantial reduction in the reporter response in the presence of a TLR3-blocking antibody.

More studies will be required for precise identification of the necrotic biomolecules behaving like TLR3 agonists. However, we already have substantial evidence that RNA molecules are the main players. There was a consistent and dramatic reduction in the effects of the conditioned media from HNSCC cells subjected to pronecrotic stimuli when these media were pretreated with RNAse prior to application on reporter cells. This is consistent with our knowledge of double-stranded RNA being the main specific PAMP ligand for TLR3 [23,24]. More specifically, this is consistent with a previous study on TLR3 ligands resulting from mechanical necrosis of HEK 293 cells. These ligands were identified as double-stranded RNAs [15]. However, we cannot formally exclude the possibility that other necrotic biomolecules are involved in TLR3 activation, especially when the effects on reporter cells are only slightly reduced by RNAse treatment, for example, for the necrotic fluid from the C17 xenografted tumor. Necrotic proteins that can bind TLR3, like HMGB1, might be involved in its activation [25].

Finally, there is one important question to determine: what are the consequences of the release of TLR3 agonists by necrotic cells for tumor growth? If we first consider the classical role of TLR3 agonist in connection with cells of innate immunity, we can expect some enhancement of the antitumoral immune response resulting from enhanced activity of innate immune cells like natural killer (NK) cells and dendritic cells [26,27,28,29]. However, this is contradictory to the fact that tumors with large amounts of necrosis are generally the most aggressive. This might be explained to some extent by the high rate of necrosis, reflecting a very rapid proliferation, with angiogenesis lagging well behind, but it might also be explained by the deleterious effects of some categories of biomolecules released by necrotic cells [6]. In this regard, it is interesting to note that TLR3 is frequently expressed by tumor cells in human malignancies. This has been reported for prostate, hepatocellular, cervical (HPV-positive) and breast carcinomas, as well as multiple myeloma and melanoma [30,31,32,33,34,35]. TLR3 is also frequently expressed by HNSCC cells, fresh tumor specimens and most HNSCC cell lines propagated in vitro. In contrast, it is generally absent in healthy tissues adjacent to tumors [36]. The influence of TLR3 expression on the tumor outcome is variable. It is associated with a positive prognosis in hepatocellular carcinomas, although this is an exception [37]. In contrast, high TLR3 expression is associated with poor prognosis in prostate, breast, lung, ovarian, gastric, esophageal and oral carcinomas [37]. This might be surprising in view of some reports about apoptosis induced in vitro by TLR3 stimulation in various cell types [38,39]. However, the proapoptotic effect of synthetic TLR3 agonists is often restricted to a limited number of cell lines and often requires very high concentrations in the range of 50 μg/mL. In contrast, we and others have shown that under various experimental conditions, TLR3 activation can also promote cancer cell metabolic reprogramming, proliferation, migration and invasiveness [40,41,42,43,44,45,46].

Overall, our data converge with published data to support the idea that necrotic biomolecules released in the tumor microenvironment by malignant cells—especially dsRNAs—contribute to tumor growth through autocrine TLR3 stimulation. This hypothesis deserves further investigations using experimental tumor models and clinical material, especially in connection with HNSCCs. To this end, it will be useful to work on syngeneic HNSCC models instead of the Renca model, which represents a renal carcinoma. Right now, it is interesting to observe that among the five patients from whom we collected samples of tumor necrotic fluid, the three samples with the highest level of stimulation on TLR3 reporter cells were from patients with very aggressive diseases presenting extranodal extension and bone invasion.

Beyond the possible involvement of TLR3 in the progression of HNSCCs, one merit of our study is that it shows that functional studies are possible on tumor necrotic fluids collected intraoperatively. This procedure, which can be performed with ordinary needles and syringes, is easy, rapid and not harmful for the patients. In the future, combinations of biochemical and functional investigations on tumor necrotic fluids might provide useful information for improved personalized treatments.

## 4. Materials and Methods

### 4.1. Cells and Reagents

Human head and neck cancer cell lines (SQ20B, FaDu and Detroit 562) were maintained in Dulbecco’s modified Eagle medium (DMEM) (Life technologies, Gaithersburg, MD, USA) supplemented with 2 mM L-glutamine and 10% fetal calf serum in a humidified chamber at 37 °C in 5% CO_2_. Detroit 562 (batch No. 70004014) and FaDu (batch No. 63372030) cell lines were obtained from the ATCC (LGC Standards). The SQ20B line was provided by Prof. Eric Deutsch (Gustave Roussy, Villejuif, France). HEKBlue^TM^ hTLR3 and HEKBlue^TM^ Null1 cells were purchased from InvivoGen (San Diego, CA, USA; batch No. S01-4101). The cells were cultured at 37 °C in 5% CO_2_ in 10 cm plastic dishes using DMEM containing glutamine, heat-inactivated fetal bovine serum (FBS), penicillin/streptomycin, and Normocin (InvivoGen). Selection of the plasmids in HEKBlue hTLR3 cells required the use of blasticidin (InvivoGen), and HEKBlue Null1 cells required the use of zeocin (InvivoGen). Cell counts and viabilities were determined by a hemocytometer and Trypan Blue exclusion. Poly(I:C) and poly(A:U) were obtained from InvivoGen (San Diego, CA, USA).

### 4.2. Cell Lines and PDX

Renca is a murine renal carcinoma cell line syngeneic with Balb-C mice (https://www.cellosaurus.org/CVCL_2174, PMID 31220119, PMID 3486710, accessed on 1 April 2023). In vitro, it was propagated in plastic flasks using RPMI 1640 culture medium with 10% FBS and 5 µg/mL gentamicin. Tumor growth in Balb-c mice was obtained by dorsal subcutaneous injections of 2 × 10^6^ cells in 200 μL PBS. Then, 10 to 15 days later, mice were sacrificed prior to tumor collection in order to keep the tumor volume below a maximum of 1.7 cm^3^. C17 and C18 tumors are patient-derived xenografts (PDX) initially grown from fragments of human metastatic nasopharyngeal carcinomas (PMID: 2971626; PMID:24618637). C17 was grown from the biopsy of a cutaneous metastasis taken from a multi-treated 38-year-old male patient. C18 was derived from the biopsy of an early lymph node metastasis with primary resistance to chemotherapy taken from a 48-year-old male patient. The C17 and C18 PDX were propagated on nude mice by iterative dorsal subcutaneous inoculation of tumor fragments: inoculation of 150 mg of tumor fragments and collection of tumors after 3 to 5 weeks when they were below the ethical threshold of 1.7 cm^3^.

### 4.3. Animal Care

Tumor expansion was conducted in female mice aged 6 to 8 weeks: immunocompetent Balb/C or nude mice (mostly of Swiss genetic background) for Renca cells and C17/C18 PDX, respectively. The animals were supplied by Janvier Labs (Le Genet-St Isle, France) and hosted in batches of five in regulatory plastic cages (27 × 22 × 15 cm) under controlled conditions (day/night cycle of 12 h, 22 ± 1 °C and 55% humidity) with water and food ad libitum. All breeding operations and experiments were conducted in accordance with a protocol validated by the Gustave Roussy ethics committee and deposited in the APAFiS platform of the French Ministry of Agriculture (Apafis #12147-201711081244492 and #31464-2021050819051109 for Renca cells on Balb/C and C17/C18 PDX on nude mice, respectively).

### 4.4. Preparation of Necrotic Material Fluids from Ex Vivo Tumors

Necrotic material, i.e., dead cells and biomolecules released by dead cells, was prepared from fragments derived from mouse experimental tumors and from clinical tumor specimens immediately or shortly after ex vivo collection. Enrichment in necrotic material was based on the mincing of tumor fragments with scissors and forceps in ordinary culture medium. In our experience, this is sufficient to trigger the release of abundant necrotic material from the large majority of solid tumor types, regardless of their anatomic origin and histology.

Experimental tumors were removed under sterile conditions from the backs of recipient mice immediately after their sacrifice by cervical dislocation to avoid prolonged tumor anoxia. They were immediately weighed and minced into fragments with diameters of about 3 mm in RPMI-1640 medium in Petri dishes (5 mL of medium for 500 mg of tumor). The residual tumor fragments were then decanted for 2 min in 15 mL Falcon tubes, and the supernatant was subjected to 500× *g* centrifugation for 7 min at 20 °C to remove dead cells and debris. The resulting clarified supernatant was then aliquoted and kept frozen at −80 °C until its use for in vitro experiments.

The same procedure for enrichment in necrotic material was applied to tumor fragments derived from surgical samples from five patients bearing oral squamous cell carcinoma and admitted to the Clinical Hospital Dubrava, Zagreb, Croatia. The collection of tumor fragments and the following procedure were performed according to ethical protocols approved by the Institutional Ethics Committee. Informed patient consent was obtained prior to surgery. The study was performed in accordance with the ethical standards laid down in the 1964 Declaration of Helsinki and its later amendments or comparable ethical standards.

### 4.5. Stimulation and HEKBlue Detection Assay

Cells were grown to 50–70% confluence and plated at a cell density of 3 × 10^4^ viable cells in 150 µL per well in a 96-well flat-bottomed tissue culture plate prior to treatment in HEKBlue detection medium (Invivogen). Then, 50 µL of supernatants from different head and neck cancer cells after different stresses was added to wells. Head and neck cancer cells were stressed as follows: serum deprivation and irradiation for 48 h and hypoxia and oxidative stress for 24 h. For serum deprivation, cells were washed twice with medium without serum and incubated with the same medium. For gamma irradiation experiments, cells were exposed to ^60^Co γ-irradiation through a panoramic source (2 Gy/min, Rudjer Boskovic Institute, Radiation Chemistry and Dosimetry Laboratory) to a total of 10 Gy. Hypoxia was performed in a hypoxia chamber according to the manufacturer’s instructions (Stemcell Technologies, Vancouver, BC, Canada). Oxidative stress was induced by the addition of 500 µM H_2_O_2_. Additionally, supernatants were incubated with 20 µg of RNAse (Sigma-Aldrich, Schnelldorf, Germany) for 2 h at 37 °C as described previously [14]. Relative NF-κB activity was determined by measuring the SEAP activity that accumulated in the culture media following overnight incubations with the supernatants. SEAP activity was measured at 620 nm using an Infinite^®^ 200 PRO microplate reader spectrophotometer (Tecan, Männedorf, Switzerland). HEKBlue-Null1 cells served as negative controls for TLR3 activation, as they should be non-responsive to TLR3 ligands. For the treatment with exosomes, 10 μg of exosomes was incubated with 150 µL of HEKBlue-TLR3 and HEKBlue-null cells (3 × 10^4^ cells) in HEKBlue detection medium and measured as cell lines. For the treatment with mice and patient aspirates, 50 µL of aspirate was incubated with 150 µL of HEKBlue-TLR3 and HEKBlue-null cells (3 × 10^4^ cells) in HEKBlue detection medium and measured as cell lines. RNAse treatment was performed with 40 µg of RNAse (Sigma) for 2 h at 37 °C for patients’ and mice supernatants or with 20 µg of RNAse (Sigma) for 2 h at 37 °C for cell supernatants. TLR3 antibody (Santa Cruz) was added at a concentration of 20 μg/mL. In all experiments, Poly(I:C) was used as a positive control at a concentration of 100 ng/mL.

### 4.6. Exosome Isolation

Cells were seeded at a density of 0.5 × 10^6^ cells. The following day, cells were washed with PBS, and the medium was replaced with a medium containing exosome-depleted FBS (Gibco). Cells were exposed to 60Co γ-irradiation from a panoramic source (2 Gy/min) for a total dose of 10 Gy. Then, 48 h later, irradiated exosomes were isolated using a PureExo^®^ Exosome Isolation kit (101 Bio, Mountain View, CA, USA) according to the manufacturer’s instructions. Proteins were quantified using a DC protein assay kit (Biorad, Hercules, CA, USA).

### 4.7. Transmission Electron Microscopy (TEM)

Purified exosome preparations (5 µL) were placed on a Formvar^®^/carbon copper grid and air-dried for 20 min. Afterwards, they were contrasted with 2% uranyl acetate for 7 min, then washed several times. The morphology of isolated exosomes was visualized with TEM (FEI MORGAGNI 268D).

### 4.8. Nanoparticle Tracking Analysis

Quantification of total particles was performed using a NanoSight LM10 instrument equipped with an sCMOS camera and a red laser (Malvern Panalytical Ltd., Malvern, UK). Nanoparticle tracking analysis (NTA) was performed on samples that were diluted at a ratio of 1:25 in qH_2_O to obtain 10–100 particles in the field of view.

### 4.9. Western Blot Analysis

Proteins were isolated with RIPA buffer and transferred onto a 0.2 µm nitrocellulose membrane as described previously [40]. The membranes were blocked with 5% nonfat dry milk and stained with primary antibodies: anti-CD63 (Abcam, Cambridge, UK), calnexin (Cell signaling technology, Beverly, MA, USA) and β-actin (Cell signaling technologies). Membranes were also stained with amido black as a loading control. Afterwards, the membranes were stained with peroxidase-conjugated secondary antibody (Cell signaling technology) and visualized with the following chemiluminescent systems: Western Lightning^®^ Plus ECL (Perkin Elmer, Waltham, MA, USA) for cellular proteins and SuperSignal™ West Atto (Thermo Scientific, Waltham, MA, USA) for exosomal proteins. Images were obtained using an Alliance Q9 mini instrument (UVitec, Cambridge, UK).

### 4.10. Statistics

Statistical significance was assessed with two-tailed Student’s *t*-test, and the results are presented as the mean ± SD.

## 5. Conclusions

Overall, our data indicate that TLR3 agonists are consistently present in necrotic fluids from HNSCC cells and tumors and are most likely mainly made of dsRNA fragments. This is an important finding because TLR3 is not always antitumorigenic. We previously reported that it can also have a protumorigenic effect in HNSCC [40,43,45]. Therefore, it is important to keep in mind that no external ligands are necessary for TLR3 stimulation and its potential contribution to cancer progression. Endogenous TLR3 ligands can be provided by the tumor itself. This is consistent with previous observations showing that necrotic tumors are more aggressive. It is also consistent with our findings that the three samples of the necrotic fluid with the highest levels of TLR3 stimulation were from patients with very aggressive cancers. In addition, we report that at least a fraction of the tumor necrotic endogenous TLR3 ligands are contained in extracellular vesicles. This means that they can be spread in various organs through the body and may possibly be involved in processes facilitating metastatic spread. Regarding methodological aspects, our study demonstrates that direct investigations, including functional testing, can be performed on necrotic fluids from patient HNSCC lesions.

## Figures and Tables

**Table 1:** Clinicopathological data about patients, tumor location, type and risk factors.

Patient No.	Sex	PHD	Tumor Localization	Tumor Differentiation	Ptnm	Age Group	Smoking	Alcohol	Hepatitis	HPV	EBV	FA/DC	Diabetes
1	M	OSCC	retromolar trigone	well (I)	T4An0M0	70–75	Yes	Yes	No	No	No	No	No
2	M	OSCC	mandibular alveolar ridge	well (I)	T4An0M0	70–75	Yes	Yes	No	No	No	No	No
3	F	OSCC	tongue	well (I)	T1N2Bm0	40–45	No	No	No	No	No	No	No
4	M	OSCC	floor of mouth	well (I)	T4An3Bm0	60–65	Yes	Yes	No	No	No	No	No
5	F	OSCC	floor of mouth	well (I)	T4An3Bm0	55–60	Yes	No	No	No	No	No	No

PCC–planocellular carcinoma; FA—Fanconi anemia; DC—dyskeratosis congenita; pTNM—postsurgical histopathological classification; OSCC—oral squamous cell carcinoma.

## Data Availability

The data presented in this study are available upon request from the corresponding author.
